# Essential Oil of *Acorus tatarinowii* Schott Ameliorates *Aβ*-Induced Toxicity in *Caenorhabditis elegans* through an Autophagy Pathway

**DOI:** 10.1155/2020/3515609

**Published:** 2020-12-22

**Authors:** Xin-yan Chen, De-chun Liao, Meng-lu Sun, Xiang-huan Cui, Hong-bing Wang

**Affiliations:** Putuo District People's Hospital, School of Life Sciences and Technology, Tongji University, Shanghai 200092, China

## Abstract

**Background:**

*Acorus tatarinowii* Schott [Shi Chang Pu in Chinese (SCP)] is a traditional Chinese medicine frequently used in the clinical treatment of dementia, amnesia, epilepsy, and other mental disorders. Previous studies have shown the potential efficacy of SCP against Alzheimer's disease (AD). Nevertheless, the active constituents and the modes of action of SCP in AD treatment have not been fully elucidated.

**Purpose:**

The aim of this study was to investigate the protective effects of SCP on abnormal proteins and clarify its molecular mechanisms in the treatment of AD by using a *Caenorhabditis elegans* (*C. elegans*) model.

**Methods:**

This study experimentally assessed the effect of SCP-Oil in CL4176 strains expressing human *Aβ* in muscle cells and CL2355 strains expressing human *Aβ* in *pan*-neurons. Western blotting, qRT-PCR, and fluorescence detection were performed to determine the oxidative stress and signaling pathways affected by SCP-Oil in nematodes.

**Results:**

SCP-Oil could significantly reduce the deposition of misfolded *Aβ* and polyQ proteins and improved serotonin sensitivity and olfactory learning skill in worms. The analysis of pharmacological action mechanism of SCP-Oil showed that its maintaining protein homeostasis is dependent on the autophagy pathway regulated partly by *hsf-1* and *sir-2.1* genes.

**Conclusion:**

Our results provide new insights to develop treatment strategy for AD by targeting autophagy, and SCP-Oil could be an alternative drug for anti-AD.

## 1. Introduction

Alzheimer's disease (AD) is an age-related neurodegenerative disorder clinically featuring loss of memory, cognitive, and behavior functions. To date, only donepezil, galantamine, memantine, and rivastigmine have been approved by the United States Food and Drug Administration for the treatment of mild to moderate AD, but these drugs cannot interrupt or halt disease progression [1]. Therefore, more efficacious therapeutic drugs are required for the management of AD. The pathogenesis of Alzheimer's disease is associated with abnormal proteins including A*β* and tau aggregation in the brain. Generally, there is no deposition of A*β* peptide in the healthy brain, but the increased level of the A*β* peptide aggregation is manifested in the AD brain. Growing studies indicated that autophagy contributes to the degradation of abnormal proteins [2]. It has been reported that many medicinal herbs contain promising autophagy regulators and have great therapeutic potential for AD treatment [3].

*Acorus tatarinowii* Schott [*A. tatarinowii*, Shi Chang Pu in Chinese (SCP)] is a renowned traditional Chinese medicine that was first recorded in the Shennong Materia Medica. It is commonly used in the clinical treatment of dementia, amnesia, epilepsy, and other mental disorders [4]. Earlier studies showed that the essential oil from SCP prevented hydrogen peroxide-induced cell injury in PC12 cells (Yan et al., 2020), and *β*-asarone, a major component of SCP, showed protection against oxidative stress and neuronal damage induced by amyloid-*β* in rats [5, 6]. However, the underlying pharmacological action mechanism of SCP-Oil remains unclear.

In this study, we first used *Caenorhabditis elegans* (*C. elegans*) as an *in vivo* model to elucidate the action mechanism of SCP-Oil. *C. elegans* is widely used in laboratory research as it is rapidly propagated and has a short life cycle, a simple structure, and extensive homology with mammals [7]. Moreover, *C. elegans* can be readily induced by gene editing to express human *Aβ* in their muscle cells and neurons, so it is considered to be a powerful model for screening AD-related drugs and clarifying their mechanisms [8].

Here, we reported the protection effects of SCP-Oil in a transgenic A*β C. elegans* model. Our results showed that it can reduce ROS accumulation and show protection effects against abnormal A*β* and polyQ proteins by targeting autophagy degradation pathway. Our findings furtherly prove the potential of SCP-Oil to be used in AD treatment.

## 2. Materials and Methods

### 2.1. SCP-Oil Extraction and Separation

*Acorus tatarinowii* Schott (SCP) was purchased from Beijing Tongrentang Co. Ltd. (Shanghai, China). 300 g dry roots was ground in a high-speed blender and extracted twice by refluxing in 3-5× petroleum ether at 80°C for 1 h. The extracts were pooled and concentrated at 50-60°C under reduced pressure. The oil extract yield was 6.27 g.

### 2.2. Strains

The following *C. elegans* strains were acquired from Caenorhabditis Genetics Center (CGC; Minneapolis, MN, USA; funded by the National Institutes of Health (NIH) Office of Research Infrastructure Programs (P40 OD010440)): CL4176[dvIs27 [myo-3p::Abeta (1-42)::let-851(3′UTR) + rol-6(su1006)] X]; CL802[smg-1(cc546) I; rol-6(su1006) II] control for CL4176. CL2355[dvIs50 [pCL45 (snb-1::Abeta(1-42)::3′UTR(long) + mtl-2::GFP] I]; CL2122[dvIs15 [(pPD30.38) unc-54(vector) + (pCL26) mtl-2::GFP] control strain for CL2355. BC12921[sIs10729 [rCes T12G3.1::GFP + pCeh361], AM140[rmIs132 [unc-54p::Q35::YFP]].

### 2.3. Strain Maintenance and Treatment

All strains were maintained at 16°C except for BC12921 and AM140 which were maintained at 20°C. The worms were cultured on solid nematode growth medium (NGM) [9] plates containing a lawn of *Escherichia Coli* (*E. coli*) OP50 and fed with the drugs from the time they were age-synchronized to adult stage.

### 2.4. High-Performance Liquid Chromatography (HPLC) of SCP-Oil

A ZORBAX Eclipse XDB C-18 column (Agilent) was used for high-performance liquid chromatography (HPLC) analysis. The operating conditions were as follows: either 10 mg/mL extract or 1.0 mg/mL standard; 1.0 mL/min flow rate; 10 *μ*L injection volume; detection wavelength set at 254 nm; and room temperature (25°C). The eluents were as follows: A (ultrapure water) and B (100% (*v*/*v*) methanol). The gradient was as follows: 0.01 min 35% B, 30 min 75% B, 45 min 85% B, 55 min 95% B, and 65 min 95% B. Prior to their injection in the HPLC, all samples were filtered through a 45-*μ*M membrane (Agilent). The analysis result was seen in Supplementary Fig. S2.

### 2.5. Safety Assessment Assay

Synchronized L1 larvae were cultured in a 96-well plate with OP50, and 1.08 mM FUdR was added by L3. Then, young adult larvae were treated with various concentrations of SCP-Oil. The live worms were observed and recorded daily until day 4. Nematodes that were stiff and unresponsive to strong light or agitation were assumed to be dead.

### 2.6. Paralysis Assay in Nematodes

Synchronized L1 larvae (>30 worms per treatment) were transferred to 35 mm culture plates containing OP50 and drugs, cultured at 16°C for 36 h, and upshifted to 23°C for transgene induction, scoring the paralyzed worms per hour after cultured at 23°C for another 24 h. Worms were considered paralyzed when they fail to move about, did not respond to platinum wire stimulus, or presented with an anterior halo [10]. The assay was repeated at least three times, and the PT_50_ (time duration in which half worms were paralyzed) was calculated [11].

### 2.7. Western Blotting

Synchronized L1 worms were cultured for 36 h at 16°C on 100 mm NGM plates containing OP50 and drugs, upshifted to 23°C, and incubated at that temperature for another 32 h. The worms were then collected in 1x phosphate-buffered saline (PBS) (8 g NaCl, 0.2 g KCl, 1.44 g Na_2_HPO_4_, and 0.24 g KH_2_PO_4_). The total protein was isolated with ice-cold radioimmunoprecipitation assay (RIPA) lysis buffer containing 1x protease inhibitor and 1x phosphatase inhibitor cocktail and identified by using a Tris-Tricine gel (each lane was loaded 40 *μ*g protein) [11]. The A*β* protein levels were detected with 6E10 monoclonal antibody (1 : 500; BioLegend). Species-specific *β*-actin was the internal control detected with mouse *β*-actin monoclonal antibody (60008-1-Ig; 1 : 2000; Proteintech®). Anti-mouse IgG HRP-linked antibody was the secondary antibody (No. 7076, 1 : 3000; Cell Signaling Technology). Blots were visualized by standard enhanced chemiluminescence (ECL; NCI4106; Thermo Fisher). The protein signals were quantified with Gel-Pro Analyzer 4.

### 2.8. Chemotaxis Learning Assay

Equal volumes (on demand) of 1 M sodium acetate and 1 M sodium azide were blended and used as an attractant. The control odorant was a mixture of 1 M sodium azide and sterile water. Synchronized CL2355 and control strain CL2122 larvae were cultured on either an untreated or drug-loaded NGM plate at 16°C until L3 (~36 h) and upshifted to 23°C for another 36 h. The worms were collected, and the OP50 with M9 Buffer (3 g KH_2_PO_4_, 6 g Na_2_HPO_4_, 5 g NaCl, 1 mL 1 M MgSO_4_, and ddH_2_O to make up 1 L) were cleared out. 40_60 worms were placed in the center of a clear 10-cm NGM plate, 10 *μ*L attractant was quickly dropped onto one side of the plate, and 10 *μ*L control odorant was dropped onto the other side [12]. After 1 h, the number of worms near each spot was recorded and the chemotaxis index was calculated as follows: chemotaxis index (CI) = (number of worms on attractant side − number of worms on the control side)/total number of worms.

### 2.9. Fluorescence Assay

Synchronized BC12921 worms were cultured in 96-well plates containing OP50. Drugs were administered at L4, and the worms on day 4 of adulthood were collected. The OP50 and drugs were replaced with M9, and the worms were transferred to a clear 35 mm NGM plate. The worms were then transferred to a black 96-well plate containing 200 *μ*L M9 buffer (10 worms/well, 60 worms/treatment). Relative fluorescence was detected and quantified in a SpectraMax® ID5 multi-mode microplate reader at 485 nm excitation and 535 nm emission.

### 2.10. ROS Assay

The CL4176 and CL802 worms were synchronized. L1 larvae were exposed to either SCP-Oil or dimethyl sulphoxide (DMSO) in 35 mm NGM plates at 16°C for 36 h and upshifted to 23°C for 32 h; the worms were harvested and washed thrice with M9 to remove the OP50 and drugs. The worms were then transferred to a black 96-well plate containing 120 *μ*L of 1% Tween-20 in M9 (15/well). Then, 8.0 *μ*L of 50 *μ*M 2,7-dichlorofluorescein diacetate (H_2_DCFDA) (Sigma) was rapidly added. The black plate was incubated at 37°C for 2 h; subsequently, the fluorescence intensity was detected every 20 min for 2 h using a SpectraMax® ID5 microplate reader with excitation/emission at 485 nm/535 nm.

### 2.11. Total RNA Isolated and qRT-PCR

Starting at L1, synchronized CL4176 worms were fed with OP50 and drugs at 16°C for 36 h and upshifted to 23°C for 32 h. The worms were then harvested, washed twice with PBS to remove the OP50 and drugs, washed 3-5 times with diethylpyrocarbonate (DEPC) sterile water, and frozen at -80°C for 2 h. Total RNA was isolated by TRIzol methods [13] and converted to cDNA with an All-in-One cDNA synthesis supermix kit (No. B24403; Bimake). Quantitative real-time PCR (qRT-PCR) was performed using a 2 × SYBR Green qPCR master mix kit (No. B21202; Bimake). The qRT-PCR operating conditions were as follows: 95°C for 5 min, followed by 40 cycles of 95°C for 15 s, and 60°C for 45 s. The melting curve was plotted under 95°C for 15 s, 60°C for 1 min, and 95°C for 15 s followed by cooling and maintenance at 4°C. *β*-Actin was the housekeeping gene. The transcription levels were analyzed by the 2^-*ΔΔ*Ct^ method. The primers used in this assay are listed in Supplementary Table S7.

### 2.12. Serotonin Sensitivity Assay

The L1 of CL2355 and its control strain CL2122 were treated with or without drugs, maintained at 16°C for 36 h, and then moved to 23°C for 36 h. The worms were collected and washed thrice in M9 buffer to remove the *E. coli* and drugs and then transferred to a 96-well-plate containing 200 *μ*L of 5 mg/mL serotonin dissolved in M9. Active worms were recorded after 24 h. At least three independent assays were conducted.

### 2.13. Statistical Analysis

At least three independent experiments were performed per assay. Results were calculated and analyzed by Student's *t*-test. *P* < 0.05 was consider statistically significant.

## 3. Results

### 3.1. SCP-Oil Retards A*β*-Induced Paralysis in *C. elegans*

**Figure 1:**
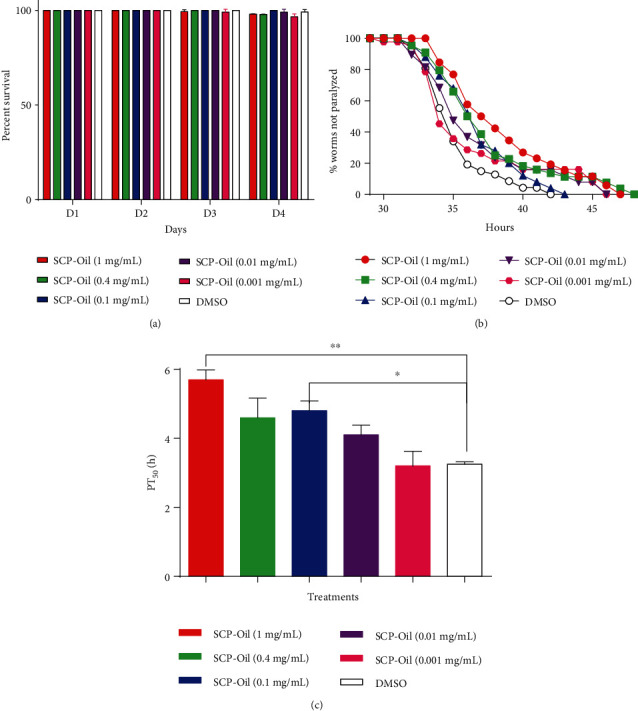
Various SCP-Oil concentrations impeded *Aβ* induced paralysis in *C. elegans*. (a) Percent survival of CL4176 at 16°C in 96-well plates containing 0, 0.001, 0.01, 0.1, 0.4, and 1 mg/mL SCP-Oil. (b) Percentage of worms not paralyzed at the various SCP-Oil concentrations. (c) PT_50_ for untreated worms and those treated with SCP-Oil. Data were analyzed by Student's *t*-test. Error bars indicate the means ± SD. ^∗∗^*P* < 0.01 and ^∗^*P* < 0.05.

Extracellular *β*-amyloid (A*β*) deposition is the main AD pathogenesis, which is neurotoxic and myotoxic [14]. To determine whether SCP-Oil protects against A*β*-induced toxicity *in vivo*, we assessed the efficacy of SCP-Oil on delaying *Aβ*-induced paralysis in transgenic CL4176 worms expressing temperature-induced human A*β* protein. We treated the worms with 0-1.0 mg/mL SCP-Oil in 96-well plates at 16°C for 4 d. The results showed that SCP‐Oil < 1.0 mg/mL had no effect on nematodes' lifespan; it was suggested that SCP‐Oil < 1.0 mg/mL was not toxic to the worms (Figure 1(a)). We then performed a paralysis assay on CL4176 worms using 0, 0.001, 0.01, 0.1, 0.4, and 1.0 mg/mL SCP-Oil. Our results showed that SCP-Oil delayed paralysis in CL4176 worms in a dose-dependent manner (Figure 1(b) and Table S1). Further, the PT_50_ for worms treated with 1 mg/mL SCP-Oil and the untreated worms were 5.70 ± 0.20 h and 3.25 ± 0.05 h, respectively; 1 mg/mL SCP-Oil significantly extended the PT_50_ by up to 75.39% relative to the untreated control (Table S1). Therefore, 1 mg/mL SCP-Oil was used in the subsequent assays. Overall, the above results suggested that none of the tested SCP-Oil concentrations was toxic to the worms and all could potentially protect them against *Aβ*-induced damage.

### 3.2. SCP-Oil Enhances Olfactory Learning and Serotonin Sensitivity in Nematodes with Neuronal A*β* Expression

**Figure 2:**
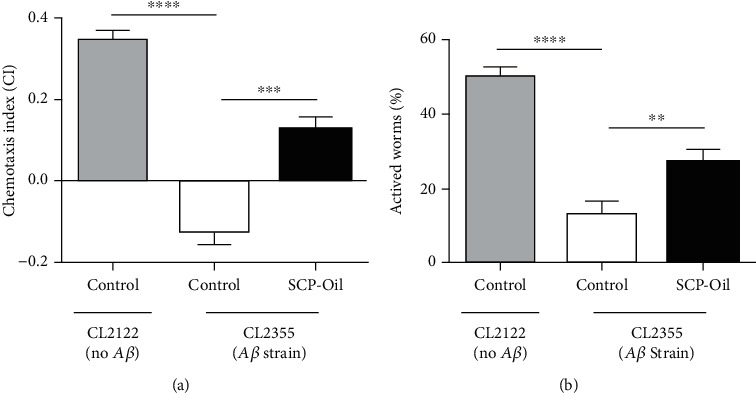
SCP-Oil enhanced chemotaxis and serotonin sensitivity in *pan*-neuronal *Aβ* transgenic worms. (a) Chemotaxis in CL2355 and its control strain CL2122. (b) SCP-Oil increased serotonin sensitivity relative to the untreated control group. Three independent experiments were conducted per assay. Values are the means ± SD. ^∗∗∗∗^*P* < 0.0001, ^∗∗∗^*P* < 0.001, and ^∗∗^*P* < 0.01.

CL2355 is a transgenic strain in which *Aβ* was expressed in their neuronal cells; it was showed deficits in chemotaxis and associative learning skills [15]. To investigate the protective effects of SCP-Oil on the neurological functions, we determined olfactory adaptation-related learning in this strain. The chemotaxis assay indicated that 1 mg/mL SCP-Oil significantly increased the number of worms on the attractant side of the plate (Figure 2(a)). The chemotaxis indexes for CL2122 control strain and the untreated CL2355 worms were 0.35 ± 0.01 and −0.13 ± 0.02, respectively (Table S2), which suggested that worms with *pan*-neuronal human *Aβ* expression did exhibit severe cognitive deficit. However, the SCP-Oil treatment significantly increased the value of CI to 0.12 ± 0.01 (Figure 2(a) and Table S2). So, SCP-Oil obviously exhibited a protective effect on the neurological functions of worms.

Neurotransmitter serotonin plays an important role in locomotion, cognition, and learning-related behavioral plasticity in *C. elegans* [16]. Here, we incubated the worms in 5 mg/mL exogenous serotonin for 24 h and recorded the number of active individuals.  Figure 2(b) shows that ~27.40 ± 1.70% of the worms were still alive after treated with 1 mg/mL SCP-Oil, but only 13.34 ± 1.50% of the control worms had survived (Table S3). Moreover, exogenous serotonin had a negligible effect on the CL2122 control (50.56 ± 1.11%; Figure 2(b) and Table S3).

Hence, 1 mg/mL SCP-Oil can obviously improve learning behavior and augment serotonergic excitability against *Aβ*-induced deficits in the neurological functions.

### 3.3. SCP-Oil Decreases A*β* Aggregation in *C. elegans*

**Figure 3:**
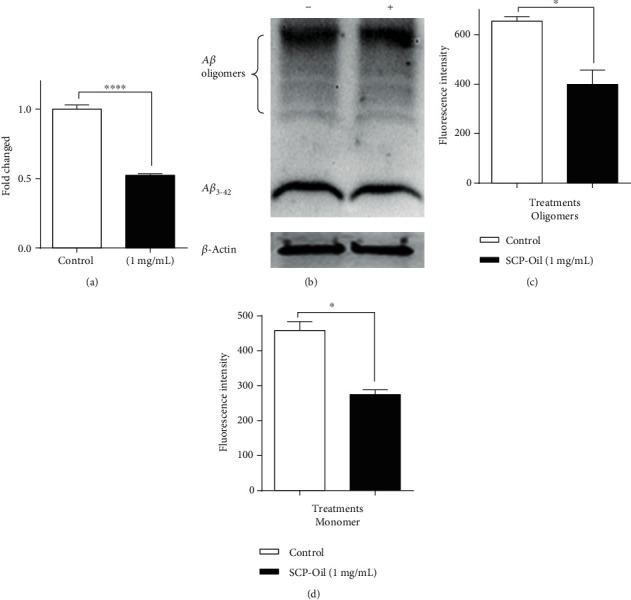
SCP-Oil decreased the *Aβ* transcript and protein levels in CL4176 nematodes. (a) The 1 mg/mL SCP-Oil treatment decreased *Aβ* expression in CL4176. (b) Western blot of A*β* oligomers and monomer in CL4176 following SCP-Oil treatment. *β*-Actin was the internal reference for normalization. (c) A*β* oligomers quantification by Gel-Pro. (d) A*β* monomer quantification in CL4176 nematodes. The assay was repeated at least thrice. Error bars represent SD. ^∗∗∗∗^*P* < 0.0001 and ^∗^*P* < 0.05.

Given that SCP-Oil could effectively delay paralysis of CL4176 worms, we evaluated the effect of SCP-Oil on *Aβ* transcription and protein levels. Relative to the untreated, the A*β* transcription level was decreased by 0.56-fold in nematodes treated with 1 mg/mL SCP-Oil (Figure 3(a)). The amyloid protein levels indicated that 1 mg/mL SCP-Oil can significantly decrease A*β* aggregation in worms; quantitative data analysis showed that A*β* oligomers and monomers were remarkably reduced by ~39.10% and 40.07%, respectively, in nematodes treated with 1 mg/mL SCP-Oil (Figures 3(b)–3(d)). Therefore, SCP-Oil resisted *Aβ*-induced damage in *C. elegans* by downregulating *Aβ* transcription and decreasing amyloid protein expression.

### 3.4. SCP-Oil Reduces polyQ Accumulation in *C. elegans*

**Figure 4:**
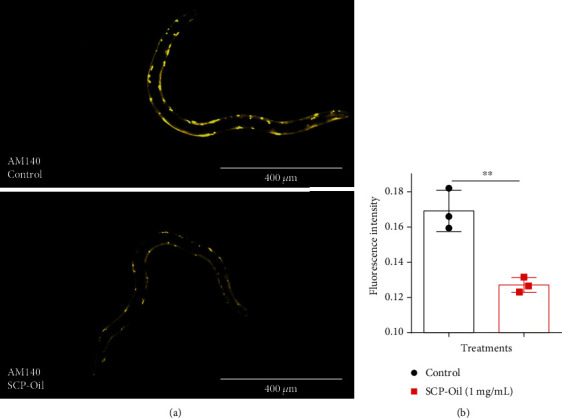
SCP-Oil decreased polyQ accumulation in AM140. (a) Representative images of AM140 [unc-54p::Q35::YFP] expressing Q35::YFP in response to no treatment or treatment with 1 mg/mL SCP-Oil. These images were taken by a fluorescence microscopy. (b) Quantification of YFP fluorescence intensity in worms either untreated or administered 1 mg/mL SCP-Oil using ImageJ 1.52a. Error bar represents the mean ± SD for ≥3 independent experiments. Significance was determined by Student's *t*-test. ^∗∗^*P* < 0.01; *n* ≥ 30.

Abnormal polyglutamine (polyQ) aggregation disrupts cellular proteostasis, triggers cytopathy, and induces neurodegenerative diseases, aggregation of polyQ protein elevating amyloidogenic processing of amyloid precursor protein by upregulating *β*-site amyloid precursor protein-cleaving enzyme 1 [17]. To determine whether SCP-Oil mitigates the accumulation of other abnormal proteins *in vivo*, we measured polyQ accumulation in AM140 strain. Fluorescence images disclosed there was less polyQ aggregation in worms treated with 1 mg/mL SCP-Oil than there was in control worms (Figure 4(a)). Quantitative data analysis revealed that the polyQ level was 24.85% lower in the treated group than it was in the control (Figure 4(b) and Table S4). The results suggested that 1 mg/mL SCP-Oil could significantly alleviate the proteotoxic stress induced by polyQ aggregation.

### 3.5. SCP-Oil Decreases A*β*-Induced ROS Accumulation in *C. elegans*

**Figure 5:**
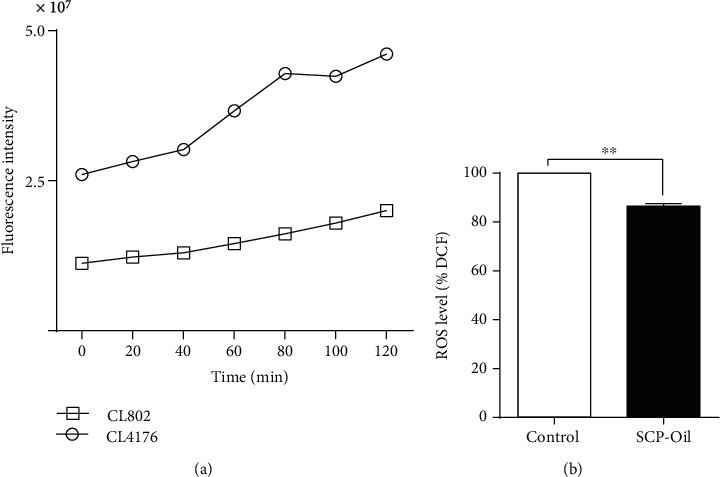
SCP-Oil mitigated *Aβ*-induced ROS accumulation in *C. elegans*. (a) The ROS fluorescence intensity in CL4176 worms and its control strain CL802 worms. (b) The 1 mg/mL SCP-Oil treatment attenuated *Aβ*-induced ROS formation in transgenic CL4176 worms. The control group fluorescence intensity was 100% and that for the treatment group was calculated relative to that of the control. Error bars represent the means ± SD. ^∗∗^*P* < 0.01; *n* ≥ 60.

A*β* aggregation causes mitochondrial dysfunction which, in turn, leads to the release of reactive oxygen species (ROS). Excessive ROS potentiates A*β* toxicity and promotes neuroinflammation [18]. We measured the ROS levels in AD worms with an H_2_DCFDA kit and found that ROS levels were relatively lower in the CL802 control (Figure 5(a)). Unlike CL4176, CL802 does not express human *Aβ* in its muscle cells. In contrast, the cellular ROS level was extremely high in untreated CL4176 (Figure 5(a)). After being treated with 1 mg/mL SCP-Oil, the intracellular ROS concentration in CL4176 was significantly reduced by 13.55% compared with the ROS level of the control group (Figure 5(b) and Table S5). Thus, SCP-Oil could mitigate ROS accumulation and ameliorate cellular damage caused by A*β*.

### 3.6. Protection Effects Provided by SCP-Oil Is Autophagy-Dependent

In order to clarify the underlying signal pathway involved in the protection effects of SCP-Oil in *C. elegans*, we selected some key transcription factors involved in aging, stress resistance, and protein homeostasis, including *daf-2* and *daf-16* that play an important role in regulating lifespan and stress resistance [19]; *skn-1*, *hsf-1*, and *sir-2.1* that participate in stress resistance and protein homeostasis [20, 21]; and *bec-1*, *vps-34*, *unc-51*, *lgg-1*, and other genes that are vital for the autophagy pathway in *C. elegans* [22].

**Figure 6:**
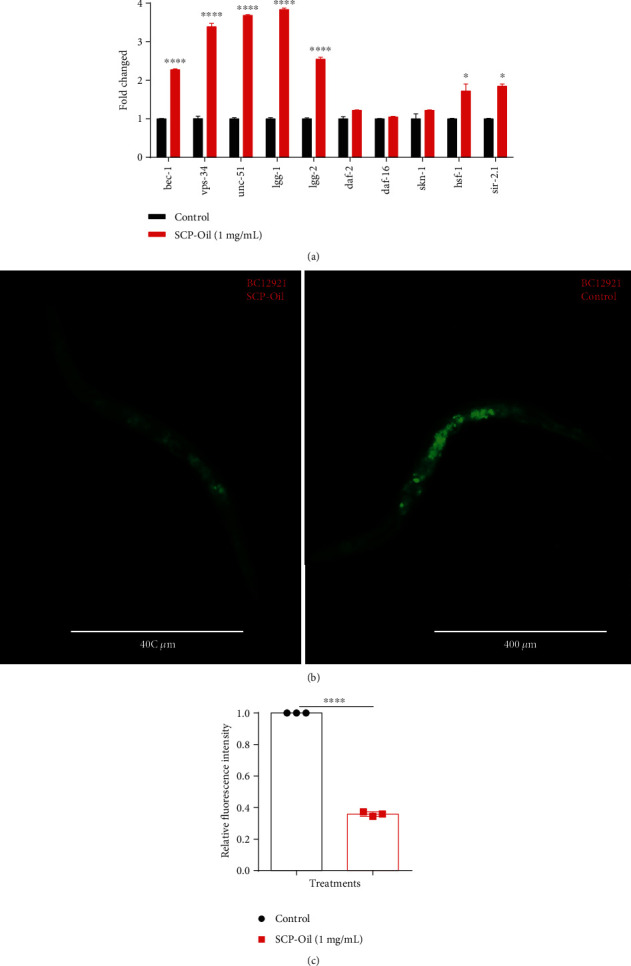
SCP-Oil protection against *Aβ-*induced damage was mediated by the autophagy pathway. (a) Gene mRNA transcription levels after treatment with 1 mg/mL SCP-Oil. (b) P62/SQSTM-1 substrate levels in BC12921 worms either untreated or treated with 1 mg/mL SCP-Oil. (c) Fluorescence intensity analysis of BC12921 cultured with or without SCP-Oil. All assays were performed at least twice. Data were analyzed by Student's *t*-test. Error bar indicates the mean ± SD. ^∗∗∗∗^*P* < 0.0001 and ^∗^*P* < 0.05.

Our results showed that SCP-Oil could not increase the expression of *daf-2* or *daf-16*, but *hsf-1* and *sir-2.1* were obviously upregulated, 1.72- and 1.85-fold higher than the control group, respectively (Figure 6(a)), that means protection effects of SCP-Oil partly depend on *hsf-1* and *sir-2.1* genes.

Moreover, the autophagy-related genes of the SCP-Oil-treated worms were dramatically upregulated. The expression levels of *bec-1*, *vps-34*, and *unc-51* were 2.28 ± 0.02-fold, 3.39 ± 0.06-fold, and 3.69 ± 0.02-fold higher, respectively, in the treated worms than they were in the control (1.00 ± 0.01) (Figure 6(a)). Besides, both *lgg-1* and *lgg-2* (homologues of mammalian LC3 associated with autophagosome and autolysosome formation) were at least 3.84-fold and 2.55-fold higher in the SCP-Oil-treated worms than they were in the control (Figure 6(a)).

P62/SQSTM1 is a ubiquitin- and LC3-binding protein and is degraded by autophagy. *In vivo*, P62/SQSTM1 accumulation is commonly accompanied by reduced autophagy [23]. The observed upregulation of autophagy-associated genes suggested that SCP-Oil could enhance autophagy activity in AD worms. We measured the P62 protein level in BC12921 stain expressing SQST-1::GFP protein. A fluorescence intensity assay demonstrated that P62 protein expression was >60% lower in the SCP-Oil-treated worms than it was in the control (Figure 6(c)). This finding indicated that autophagy was very active in nematodes treated with SCP-Oil. The above results strongly indicated that 1 mg/mL SCP-Oil showing protective effect against *Aβ*-induced injury was dependent on autophagy pathway.

## 4. Discussion

The pathogenic mechanisms of Alzheimer's disease (AD) involve the deposition of abnormally misfolded proteins, amyloid *β* protein (A*β*), and tau protein. A*β* comprises senile plaques, and tau aggregates form neurofibrillary tangles, both of which are hallmarks of AD. Although it was reported that *β*-asarone, a major component of SCP, showed protection against neuronal damage induced by amyloid-*β* in rats [5, 6], the underlying molecular mechanism of SCP is still unclear. Here, our experiment results showed that SCP-Oil can remarkably ameliorate *Aβ*-induced paralysis in worms expressing *Aβ* protein in their muscle cells compared with DMSO control or vehicle control (Figures 1(b) and 1(c) and S1). An immunoassay disclosed that SCP-Oil decreases A*β* oligomers and monomer protein levels (Figures 3(b)–3(d)), and the A*β* transcription level was significantly decreased in nematodes treated with 1 mg/mL SCP-Oil (Figure 3(a)). The above results furtherly confirmed that SCP-Oil is the main component of SCP and has an inhibitory effect on the toxicity of A*β* protein. Additionally, we found that SCP-Oil improves chemotaxis-related learning and serotonin-associated excitability in worms expressing *Aβ* in neuron cells (Figure 2), which suggested that SCP-Oil has a protective effect on neuron damage induced by *Aβ* protein. Moreover, SCP-Oil can significantly reduce the deposition of misfolded polyQ protein in AM140 strains (Figure 4). These results suggested that SCP-Oil has the effect of maintaining the homeostasis of misfolded proteins and shows the potential to develop a therapeutic for AD.

Oxidative stress has been recognized as a contributing factor in the progression of multiple neurodegenerative diseases including AD [24]. Abnormal proteins in turn could exacerbate ROS production (Figure 5(a)), thereby contributing to a vicious cycle. We found that SCP-Oil treatment substantially reduced ROS levels in CL4176 strains (Figure 5(b) and Table S5). Generally, increased production of ROS is associated with loss of mitochondrial function. *Sir-2.1*, a key gene for controlling mitochondrial function [25], was significantly upregulated (Figure 6(a)). Therefore, SCP-Oil exerting its protective efficacy against *Aβ-*induced injury partly depends on regulating mitochondrial function to decrease the level of ROS *in vivo*.

Autophagy, the main conserved pathway for the degeneration of aggregated proteins, A*β*, tau, and dysfunctional organelles in the cell, has been discovered to be involved in the pathological changes of AD [3]. Due to the fact that SCP-Oil could significantly reduce the expression of A*β* and polyQ proteins in worms, we inferred that autophagy may make a contribution for this beneficial effect of SCP-Oil. According to the results of autophagy-related genes assay, the mRNA levels of key genes (*lgg-1*, *lgg-2*, *bec-1*, *vps-34*, and *unc-51*) were remarkably upregulated (Figure 6(a)). Moreover, P62 protein, one of the best-known autophagic substrates, was obviously reduced after treatment with SCP-Oil (Figure 6(c) and Table S6). These findings suggested that the autophagy pathway was involved in the protection against abnormal A*β* and polyQ protein-induced toxicity.

Autophagy is induced in multiple tissues of *Caenorhabditis elegans* following HSF-1 overexpression, and downregulation of HSF-1 activity exacerbates misfolded and unfolded protein aggregation [26]. Our present study demonstrated that SCP-Oil significantly upregulated the *hsf-1* transcription level (Figure 6(a)). Additionally, *sir-2.1* not only participates in oxidative stress but also induces autophagy activity to reduce abnormal protein aggregation and toxicity [27]. Taken together, our results revealed that the effect of SCP-Oil maintaining protein homeostasis is dependent on the autophagy pathway regulated partly by *hsf-1* and *sir-2.1* genes.

## 5. Conclusions

In summary, our results confirmed that SCP-Oil is the main component of SCP showing a protection effect against abnormal proteins in worms. We further clarified the pharmacological action mechanism of SCP-Oil and showed that it reduces A*β* and polyQ deposition through the targeting autophagy pathway. In the recent few years, much progress has been made in finding autophagy regulators from natural products, which provides new insights to develop treatment strategy for AD by targeting autophagy. SCP-Oil could be an alternative drug for anti-AD.

## Data Availability

It can be found in Supplementary Materials file.

## Abbreviations

AD: Alzheimer's disease
A*β*: Beta-amyloid peptide
*C. elegans*: *Caenorhabditis elegans*
CGC: Caenorhabditis Genetics Center
CI: Chemotaxis index
DEPC: Diethylpyrocarbonate
*E. coli*: *Escherichia coli*
GFP: Green fluorescent protein
DMSO: Dimethyl sulphoxide
H_2_DCFDA: Dichlorofluorescein diacetate
HPLC: High-performance liquid chromatography
NGM: Nematode growth medium
PBS: Phosphate-buffered saline
PolyQ: Polyglutamine
qPCR/qRT-PCR: Real-time quantitative polymerase chain reaction
ROS: Reactive oxygen species
SCP: *Acorus tatarinowii* Schott
SCP-Oil: Essential oil of *Acorus tatarinowii* Schott.

## Supplementary Materials

Supplementary Materials The supplementary files comprise tabulated data for the paralysis, ROS, polyQ, and other assays. Table S1: SCP-Oil ameliorated A*β*-induced paralysis in C. elegans. Table S2: SCP-Oil enhanced chemotaxis behavior in CL2355. Table S3: SCP-Oil improved 5-HT sensitivity in CL2355. Table S4: SCP-Oil reduced polyQ aggradation in AM140. Table S5: SCP-Oil decreased the ROS level in CL4176. Table S6: the effects of SCP-Oil on autophagy activity in BC12921. Table S7: primer sequences used in Q-PCR. Fig. S1: the neuroprotection of SCP-Oil on A*β*-induced or non-A*β*-induced toxicity in C. elegans. Fig. S2: comparison of chromatograms of SCP-Oil and standard.
