# Supplementary material for: Essential Oil of Acorus tatarinowii Schott Ameliorates Aβ-Induced Toxicity in Caenorhabditis elegans through an Autophagy Pathway
Source: Oxid Med Cell Longev. 2020 Dec 22;2020:3515609. doi: 10.1155/2020/3515609 (PMC7773457; doi:10.1155/2020/3515609)
Supplement: Supplementary Materials — The supplementary files comprise tabulated data for the paralysis, ROS, polyQ, and other assays. Table S1: SCP-Oil ameliorated Aβ-induced paralysis in C. elegans. Table S2: SCP-Oil enhanced chemotaxis behavior in CL2355. Table S3: SCP-Oil improved 5-HT sensitivity in CL2355. Table S4: SCP-Oil reduced polyQ aggradation in AM140. Table S5: SCP-Oil decreased the ROS level in CL4176. Table S6: the effects of SCP-Oil on autophagy activity in BC12921. Table S7: primer sequences used in Q-PCR. Fig. S1: the neuroprotection of SCP-Oil on Aβ-induced or non-Aβ-induced toxicity in C. elegans. Fig. S2: comparison of chromatograms of SCP-Oil and standard. [file 3515609.f1.docx]

Essential Oil of *Acorus tatarinowii* Schott ameliorates *Aβ*-induced toxicity in *Caenorhabditis elegans* through an autophagy pathway

Xin-yan Chen^1^, De-chun Liao^1^, Meng-lu Sun, Xiang-huan Cui*, Hong-bing Wang*

Putuo District People's Hospital, School of Life Sciences and Technology, Tongji University, Shanghai 200092, China

^1^ These authors contributed equally to this work

* Corresponding authors

E-mail address: hbwang@tongji.edu.cn (H. B. Wang); cuixh@tongji.edu.cn (X. H. Cui)

**Table S1 SCP-Oil ameliorated *Aβ*-induced paralysis in *C. elegans***

| **Treatment** | **SCP-Oil**  **(1 mg/mL)** | **SCP-Oil**  **(0.4 mg/mL)** | **SCP-Oil**  **(0.1 mg/mL)** | **SCP-Oil**  **(0.01 mg/mL)** | **SCP-Oil**  **(0.001 mg/mL)** | **DMSO** |
| --- | --- | --- | --- | --- | --- | --- |
| number | 82 | 81 | 82 | 77 | 82 | 76 |
| PT_50_ | 5.70 ± 0.20 | 4.60 ± 0.40 | 4.80 ± 0.20 | 4.10 ± 0.20 | 3.20 ± 0.30 | 3.25 ± 0.05 |
| PT_50_ extension percentage (%) | 75.39%** | 41.53% | 47.69%* | 26.15% | - | - |
| *p*-value | 0.007 | - | 0.017 | - | - | - |

*p* values were calculated using the log-rank test. Values are presented as means ± SD. ***p* < 0.01, **p* < 0.05.

**Table S2 SCP-Oil enhanced chemotaxis behavior in CL2355**

| **Treatment** | **Concentration（mg/mL）** | **CI** | ***p*-value** |
| --- | --- | --- | --- |
| Control | 0 | -0.13 ± 0.02 | < 0.0001**** |
| SCP-Oil | 1 | 0.12 ± 0.01 | 0.0005** |
| **CL2122** | | | |
| Control | 0 | 0.35 ± 0.01 | - |

Data were analyzed by Student’s *t*-test. Values are means ± SD. *****p* < 0.0001, ***p* < 0.01.

**Table S3 SCP-Oil improved 5-HT sensitivity in CL2355**

| **Treatment** | **Concentration（mg/mL）** | | **Actived worms (%)** | | ***p*-value** |
| --- | --- | --- | --- | --- | --- |
| Control | 0 | | 13.34± 1.50 | | < 0.0001**** |
| SCP-Oil | 1 | | 27.40 ± 1.70 | | 0.004** |
| **CL2122** | | | | | |
| Control | | 0 | 50.56± 1.11 | - | |

Data were analyzed by Student’s *t*-test. Values are means ± SD. *****p* < 0.0001, ***p* < 0.01.

**Table S4 SCP-Oil reduced polyQ aggradation in AM140**

| **Treatment** | **Concentration（mg/mL）** | **Relative Fluorescence Intensity** | **Decreased percentage** | ***p-*value** |
| --- | --- | --- | --- | --- |
| Control | 0 | 0.169 ± 0.006 | - | - |
| SCP-Oil | 1 | 0.127 ± 0.002 | 24.85% | 0.0042**** |

Data were analyzed by Student’s *t*-test. Values are means ± SD. *****p* < 0.0001.

**Table S5 SCP-Oil decreased the ROS level in CL4176**

| **Treatment** | **Concentration（mg/mL）** | **Relative Fluorescence intensity（%）** | ***p*-value** |
| --- | --- | --- | --- |
| Control | 0 | 100 | - |
| SCP-Oil | 1 | 86.45 ± 0.74 | 0.003** |

Data were analyzed by Student’s *t*-test. Values are means ± SD. ***p* < 0.01.

**Table S6 The effects of SCP-Oil on autophagy activity in BC12921**

| **Treatment** | **Concentration（mg/mL）** | **Relative Fluorescence Intensity** | ***p-*value** |
| --- | --- | --- | --- |
| Control | 0 | 1 | - |
| SCP-Oil | 1 | 0.36 ± 0.01 | <0.0001**** |

Data were analyzed by Student’s *t*-test. Values are means ± SD. *****p* < 0.0001.

**Table.S7 Primer sequences used in Q-PCR**

| gene | | Forward primer(5'-3') | | Reverse primer(5'-3') |
| --- | --- | --- | --- | --- |
| *actin-1* | CCAGAAGAGCACCCAGTC | | TGATGTCACGGACGATTT | |
| *Aβ* | CAGAATTCCGACATGACTCAGGATATGAAG | | CCCACCATGAGTCCAATGATTGC | |
| *daf-2* | GCCCGAATGTTGTGAAAACT | | CCAGTGCTTCTGAATCGTCA | |
| *daf-16* | *ATCGTGTGCTCAGAATCC* | | *ATGAATATGCTGCCCTCC* | |
| *bec-1* | TTGAAGAAATTGTTGGCTGAGG | | AACTTCTGCATATTGACGTTCG | |
| *vps-34* | TCATCCGGCAGTTCGTGCATATTC | | TCAGGCAGTTGTTGACCTTGTTCG | |
| *unc-51* | CGCCGGTGGTTCAGCGGATT | | TATCCTGGGTGTCGGCGGGG | |
| *lgg-1* | GCCGAAGGAGACAAGATCCG | | GGTCCTGGTAGAGTTGTCCC | |
| *lgg-2* | GCCGTTTTCCATCACAATTCTA | | CACGAAAACGTAAGCGAATTTG | |
| *skn-1* | CACGCCGTCAGCGAAGTA | | ATGCTCGGTGAGTATTGG | |
| *hsf-1* | TCCGAAAGATGACTCCAC | | ACGCATCTCTGCCATTAC | |
| *sir-2.1* | TGGCTGACGATTCGATGGAT | | ATGAGCAGAAATCGCGACAC | |





**Fig. S1 The neuroprotection of SCP-Oil on *Aβ*-induced or non-*Aβ*-induced toxicity in *C. elegans*.** SCP-Oil significantly retarded *Aβ*-induced injury in CL4176 but have no effect on its control strain CL802. The assay was performed at least three independent trials.


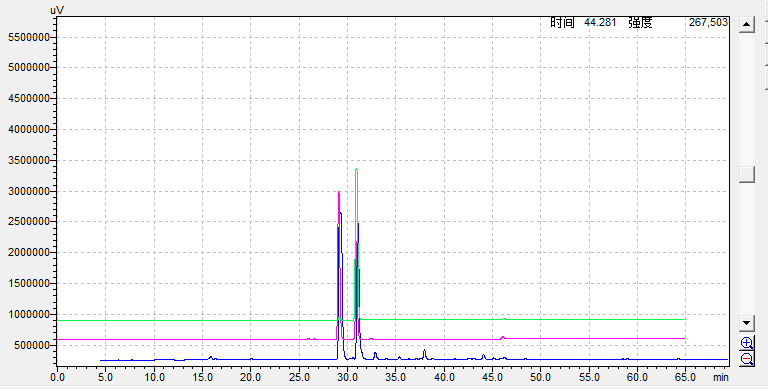


**Fig. S2 Comparison of chromatograms of SCP-Oil and standard.** Compared SCP-Oil with the standard product of α-asarone and β-asarone by HPLC (peak area percentage: α-asarone, 38.05%; β-asarone, 54.20%). Methanol as the organic phase and ultra-pure water as inorganic, 1.0 mg/mL sample, 1.0 mL/min flow rate,10 μL injection volume, detection wavelength was set at 254 nm. SCP-Oil, the blue line; α-asarone, the green line; β-asarone, the rose line.
